# Establishing a prediction model for recurrence of condyloma acuminatum

**DOI:** 10.1186/s40001-022-00816-7

**Published:** 2022-09-22

**Authors:** Mengyun Zhan, Zhenzhen Tong, Song Chen, Yu Miao, Yun Yang

**Affiliations:** 1 Department of Dermatology, Taikang Tongji (Wuhan) Hospital, Wuhan, 430050 China; 2 Department of Cardiovascular Surgery, Zhongnan Hospital of Wuhan University, Wuhan, 430071 China; 3 Department of Dermatology, The First Affiliated Hospital of Harbin Medical University, Harbin, 150001 China

## Abstract

We collected the clinical data of 156 patients diagnosed with condyloma acuminatum (CA), including age, gender, marriage, education level, stay up late, smoking, drinking, number of sexual partners, HPV infection status of sexual partners, genitourinary and anal diseases, condom use, other diseases of HPV infection, location and number of warts, HPV typing, etc. Analyze the risk factors affecting the recurrence of CA, explore the influencing factors and independent influencing factors of CA recurrence, establish the prediction model of CA recurrence, and evaluate its prediction value. Univariate analysis showed that stay up late, HPV infection status of sexual partners, urogenital diseases, condom use, other diseases of HPV infection and the number of CA were the influencing factors of CA recurrence. Multivariate analysis showed that condom use (OR = 0.166), HPV infection status of sexual partners (OR = 4.848), number of warts (OR = 1.212) and urogenital diseases (OR = 3.179) were independent factors affecting the recurrence of CA (*P* < 0.05). Therefore, the prediction model of CA recurrence can be established, and the area under the curve AUC of the prediction model was calculated to be 0.867 (95% CI 0.812–0.923). The model established in this study has certain prediction value for the recurrence of CA and can be used to preliminarily predict the recurrence of CA.

## Introduction

Condyloma acuminatum (CA) is one of the most common sexually transmitted diseases in the world caused by human papillomavirus (HPV) [1]. CA usually presents as papillomas scattered in the external genitalia, perineum, perianal and adjacent areas, and the lesions can also extend to the vagina, urethra and anal canal [2]. The infection rate of HPV is rising all over the world, reaching 11.7% [3], while 90% of CA is caused by HPV-6 and HPV-11 infection [4]. The recurrence of CA brings difficulties to the diagnosis and treatment of clinicians and causes heavy psychological pressure to patients, usually manifested as inferiority complex, tension, panic, anxiety, depression and so on [5]. The main purpose of treatment for CA is to remove visible warts, including physical removal and drug removal [6]. The primary clearance rate of these treatments is close to 100%, but the late recurrence rate is as high as 42.67% [7]. Therefore, the recurrence rate of CA has become one of the most difficult problems of this disease. In recent years, studies on recurrent CA have shown that individual differences (age, gender, marriage, cultural level, bad living habits, sexual life, etc.). HPV-related factors and wart factors are the factors affecting the recurrence of CA [8, 9]. HPV infection can also lead to other diseases, including flat warts, filamentous warts, finger/toe warts and verrucous epidermal hyperplasia in addition to CA [10]. Because they are infected by the same virus, it is worth paying attention to whether the repeated attack of CA is related to the wart infected by HPV in other parts of the body. Repeated attacks of CA affect the physical and mental health of patients, resulting in inferiority complex, tension, panic, anxiety, depression and so on [7]. Relapse prevention treatment is time-consuming, laborious and expensive. Many patients are unwilling to accept relapse prevention treatment because of economic, time and other factors. The study found that the recurrence rate of CA after wart removal treatment is between 35–75%. The span of these recurrence rates is large, which aroused our thinking. Can we establish a prediction tool based on the existing clinical data of patients, and use this tool to predict the recurrence probability of newly diagnosed CA patients after warts removal treatment? Therefore, this study took patients diagnosed with CA for the first time as the research object, based on the general data (age, gender, marriage, education level), individual situation (stay up late, smoking, drinking, sexual partners, genitourinary diseases, condom use, etc.), wart characteristics (HPV classification, location and number of CA) and other potential predictors, to establish the prediction model of late recurrence in patients diagnosed with CA for the first time.

## Materials and methods

### Study patients

This study collected three groups of data from two clinical centers. Dataset 1 included 112 patients with CA first diagnosed at the Department of Dermatological, the First Affiliated Hospital of Harbin Medical University from December 2018 to June 2020. Dataset 2 included 44 patients with CA first diagnosed at the Department of Dermatological, Taikang Tongji (Wuhan) Hospital from June 2021 to December 2021. Dataset 3 included 53 patients with CA were first diagnosed at the Department of Dermatological, the First Affiliated Hospital of Harbin Medical University from June 2020 to May 2021. The patients in dataset 1 and dataset 2 were divided into non-recurrence group and recurrence group according to the follow-up data, including 72 cases in non-recurrence group and 84 cases in recurrence group. The dataset 3 was used as the validation data. Similarly, according to the follow-up data, the patients were divided into 24 cases in the non-recurrence group and 29 cases in the recurrence group.

### Inclusion criteria

Refer to the Chinese Guidelines for Clinical Diagnosis and Treatment of Condyloma Acuminatum (2021 edition) [7]: (1) have a history of direct or indirect CA exposure. (2) Papule like, lump like, cauliflower like, chicken coronal or papillary vegetations appear in vulva, genitalia, cervix, anus and anal canal. The color is pink, crimson, gray or brown black, and the diameter of a single wart is < 3 cm. (3) Atypical patients can be diagnosed if they meet one of the following examinations: i. positive result in aceto-whitening test; ii. pathological examination: papillary hyperplasia, hyperkeratosis, dyskeratosis, epidermal acanthosis, basal cell proliferation, superficial dermal vasodilation, accompanied by inflammatory cell (mainly lymphocyte) infiltration. In the superficial layer of epidermis, koilocytosis can be observed; some keratinocytes contain deeply stained clumps of different sizes, namely virus inclusion bodies. iii. HPV nucleic acid test: the results showed HPV infection. In this study, the histopathological examination was the gold standard examination for atypical condyloma acuminata.

### Exclusion criteria

Incomplete data and missing follow-up; (2) patients who refuse or cannot cooperate; (3) patients with important organ dysfunction, malignant tumors and chronic autoimmune diseases; (4) patients with previous or suspected diagnosis of CA.

### Treatment methods

The patients included in the study were treated in the same therapy. After routine disinfection of the skin, lidocaine cream was used for local infiltration anesthesia on the wart body and surrounding skin, and carbon dioxide laser was used to remove the wart. The treatment scope reached the superficial dermis. After treatment, the wound was treated with mupirocin ointment 3 times every day until the wound healed. Apply imiquimod cream within 2 cm around the wound before going to bed every day, wash off the medicine 6–10 h after application, and use it continuously for 8 weeks. After treatment, patients should avoid scratching, reduce unnecessary activities, pay attention to rest, and avoid all kinds of CA infection pathways (such as sexual contact, toilet, bath contact, etc.).

### Observational indexes

Recurrence refers to the occurrence of new warts within 2.0 cm of the original wart site or the original wart site within 6 months after the removal of wart body treatment [11]. The histopathological examination wart body is positive, and the history of repeated infection is excluded. According to whether it recurred or not, it was divided into recurrence group and non-recurrence group.

### Investigation method

Through direct one-to-one questionnaire survey, collect the patients who meet the inclusion criteria: (1) general data: age, gender, marriage, education (highest education); (2) individual information (all in the last 3 months before the onset): age, gender, marriage, education level, stay up late, smoking, alcoholism, HPV infection status of sexual partners, genitourinary and anal diseases, condom use, location and number of warts, HPV classification, whether combined with other diseases of HPV infection. All the patients with condyloma acuminatum diagnosed in this study were examined for their sexual partners free of charge to ensure the accuracy of the questionnaire. Those who cannot cooperate with the examination have been excluded from the study.

### Statistical analysis

T-test was used to compare the age and the number of CA warts between the two groups. Chi-square test was used to compare gender, marriage, stay up late, smoking, education, sexual partners, condom use, genitourinary diseases, other diseases of HPV infection, location of CA, HPV typing and other indicators. Univariate analysis was used to determine whether the differences between the variables of the two groups were statistically significant. The variables with statistical significance were selected for univariate and multivariate binary logistic regression analysis. According to the results of multivariate binary logistic regression analysis, a model for predicting the recurrence of CA was constructed, and its statistically significant clinical variables were drawn into the receiver operating characteristic curve (ROC), and the area under the curve (AUC) was calculated. On the ROC, we found the maximum value of the sum of sensitivity and specificity according to the Youden index as the cut-off point, so as to find the patient information corresponding to the coordinates of this cut-off point, and established the discrimination equation. All statistical analyses were performed with SPSS 22.0 and a p value < 0.05 was considered statistically significant.

## Results

### Analysis of clinical indicators related to patients in two groups

A total of 156 patients with CA were included in the study. There were 84 cases (53.8%) in the recurrence group and 72 cases (46.2%) in the non-recurrence group. T-test and Chi-square test showed that gender, age, marital status, education, smoking and drinking had no effect on the recurrence of CA (*P* > 0.05), stay up late increases the recurrence of CA (P < 0.05, Table 1).

**Table 1:** Analysis of basic data of CA recurrence group and non-recurrence group

Variables	Recurrence group (*n* = 84)	Non-recurrence group (*n* = 72)	χ^2^	*P*
Gender, *n* (%)				
Male	51 (60.7)	42 (58.3%)	4.000	0.261
Female	33 (39.3)	30 (41.7%)		
Age/years, *n* (%)				
Average/median	40.94 ± 13.65	43.67 ± 12.54	–	0.833
(Range)	16 ~ 70	19 ~ 74		
≤ 30	19 (22.6)	13 (18.1)		
31–50	37 (44.1)	37 (51.4)		
≥ 50	28 (33.3)	22 (30.5)		
Marital status, *n* (%)				
Married	43 (51.2)	33 (45.8)	0.445	0.505
Unmarried	41 (48.8)	39 (54.2)		
Educational level, n (%)				
Bachelor degree or above	24 (28.6)	13 (18.1)	4.267	0.118
High school	23 (27.4)	30 (41.7)		
Middle school and below	37 (44.0)	29 (40.3)		
Smoke, n (%)				
Yes	39 (46.4)	30 (41.7)	0.356	0.551
No	45 (53.6)	42 (58.3)		
Drink, n (%)				
Yes	50 (51.0)	34 (53.7)	0.125	0.724
No	44 (49.0)	38 (46.3)		
Stay up late, n (%)				
Yes	57 (67.9)	26 (36.1)	15.693	< 0.001
No	27 (32.1)	46 (63.9)		

Sexual partner infection with HPV, condom use, number of warts, comorbid genitourinary disorders and other diseases with HPV infection had an effect on the recurrence of CA (*P* < 0.05). There were no significant differences between the two groups in the position of the warts of CA and the HPV typing (*P* ≥ 0.05, Table 2).

**Table 2:** Individual situation analysis

Variables	Recurrence group (*n* = 84)	Non-recurrence group (*n* = 72)	χ^2^	*P*
Sexual partner with HPV infection, *n* (%)				
Yes	63 (75.0)	26 (36.1)	23.929	< 0.001
No	21 (25.0)	46 (63.9)		
Condom use, *n* (%)				
Yes	22 (26.2)	45 (62.5)	20.860	< 0.001
No	62 (73.8)	27 (37.5)		
Number of warts, *n* (%)				
Average number of warts	5.3 ± 3.5	3.3 ± 3.0	–	< 0.001
≤ 3	18 (21.4)	50 (69.5)		
4–10	55 (65.5)	17 (23.6)		
≥ 10	6 (7.1)	5 (6.9)		
Complicated with genitourinary diseases, *n* (%)				
Yes	32 (38.1)	8 (11.1)	14.806	< 0.001
No	52 (61.9)	64 (88.9)		
Bacterial vaginitis	7 (8.3)	4 (5.5)		
Candida vaginitis	1 (1.2)	1 (1.4)		
Gonorrhea	4 (4.8)	1 (1.4)		
Pelvic effusion	4 (4.8)	0 (0)		
Urethritis	5 (5.8)	0 (0)		
Prepuce balanitis	11 (13.2)	2 (2.8)		
Other diseases with HPV infection, *n* (%)				
Yes	27 (32.1)	5 (6.9)	15.098	< 0.001
No	57 (67.9)	67 (93.1)		
Filamentous wart	11 (13.1)	3 (4.1)		
Flat Wart	9 (10.6)	1 (1.4)		
Verruca vulgaris	2 (2.4)	1 (1.4)		
Finger wart	3 (3.6)	0 (0)		
Toe wart	2 (2.4)	0 (0)		
The location of the wart, n (%)				
Vulva	39 (46.4)	24 (33.3)	2.761	0.097
Intra-anal or intravaginal	45 (53.6)	48 (66.7)		
Typing of HPV, *n* (%)				
High-risk type	16 (19.0)	22 (30.6)	5.972	0.05
Low-risk type	35 (41.7)	34 (47.2)		
Hybrid	33 (39.3)	16 (22.2)		

In summary, the univariate analysis showed that HPV type and location of age, sex, marriage, education, smoking, alcoholism and warts were not clinical factors associated with CA recurrence. Stay up late, sexual partners with HPV infection, condom use, genitourinary disorders, other diseases of HPV infection, and the number of warts were clinical factors associated with CA recurrence.

### Univariate and multivariate logistic regression analyses

Univariate logistic regression analysis showed that sexual partners with HPV infection, the number of warts, the use of condoms, genitourinary diseases and other diseases with HPV infection were the risk factors of CA recurrence (*P* < 0.05, Table 3).

**Table 3:** Univariate logistic regression analysis of CA recurrence

Variables	β	SE	OR	*P* value
Condom use	− 1.729	0.352	0.177 (0.089–0.354)	< 0.001
Stay up late	0.177	0.339	1.193 (0.614–2.318)	0.602
Sexual partner with HPV infection	1.669	0.352	5.038 (2.664–10.575)	< 0.001
Number of warts	0.218	0.065	1.243 (1.094–1.412)	< 0.001
Other diseases with HPV infection	1.848	0.519	6.347 (2.295–17.558)	< 0.001
Complicated with genitourinary diseases	1.594	0.437	4.923 (2.090–11.597)	< 0.001
Typing of HPV (1)	− 0.347	0.407	0.706 (0.318–1.570)	0.394
Typing of HPV (2)	0.695	0.388	2.004 (0.936–4.289)	0.074

HPV typing classification variable assignment (low-risk type: 0; high-risk type: 1; high-risk mixed type: 2)

Multivariate logistic regression analysis showed that the HPV infection status of sexual partners (OR = 4.848), the number of CA warts in patients (OR = 1.212), condom use (OR = 0.166), and concomitant urogenital disorders (OR = 3.179) were the independent influencing factors for CA recurrence (*P* < 0.05, Table 4). It could be assumed that when a CA patient had a large number of warts, accompanied by genitourinary diseases, and sexual partners with HPV infection, the possibility of CA recurrence was greater. The correct use of condoms could prevent the recurrence of CA.

**Table 4:** Multivariate logistic regression analysis of CA recurrence

Variables	β	SE	OR	*P* value
Sexual partner with HPV infection	1.579	0.426	4.848 (2.101–11.184)	< 0.001
Number of warts	0.189	0.070	1.212 (1.056–1.390)	0.006
Other diseases with HPV infection	1.111	0.585	3.037 (0.965–9.556)	0.058
Complicated with genitourinary diseases	1.156	0.525	3.179 (1.136–8.891)	0.028
Condom use	− 0.749	0.427	0.166 (0.072–0.385)	< 0.001
Constant term	− 1.201	–	–	–

### Establishing the prediction model

The results of multivariate logistic regression analysis showed that sexual partners with HPV infection, the number of warts and condom use can be used as independent influencing factors in predicting CA recurrence. According to the results of multivariate logistic regression analysis, the following predictive model could be derived. The equation is expressed as follows:

$${\text{Logit }}\left( P \right)\, = \, - {1}.{2}0{1}\, + \,{1}.{579}*{\text{HPV infection in sexual partners}}\, + \,\,0.{192}*{\text{number of warts}}\, + \,\left( { - {1}.{794}*{\text{condom use}}} \right)\, + \,\,{1}.{156}*{\text{genitourinary infection,}}$$

where Logit (*P*) was equal to ln (p/(1-p)), and *P* was the probability of recurrence. *P* = elogit (*P*)/(1 + elogit (*P*)), e represented the base of natural logarithm, and the value was about 2.718. Significance of assignment: the entry value of sexual partner with HPV infection was 1, and the entry value of sexual partner without HPV infection was 0; The input value was taken as 1 when the condom was used correctly, and 0 when the condom was not used correctly; The substitution value for patients with genitourinary diseases was 1, and the substitution value for patients without genitourinary diseases was 0.

**Fig. 1:**
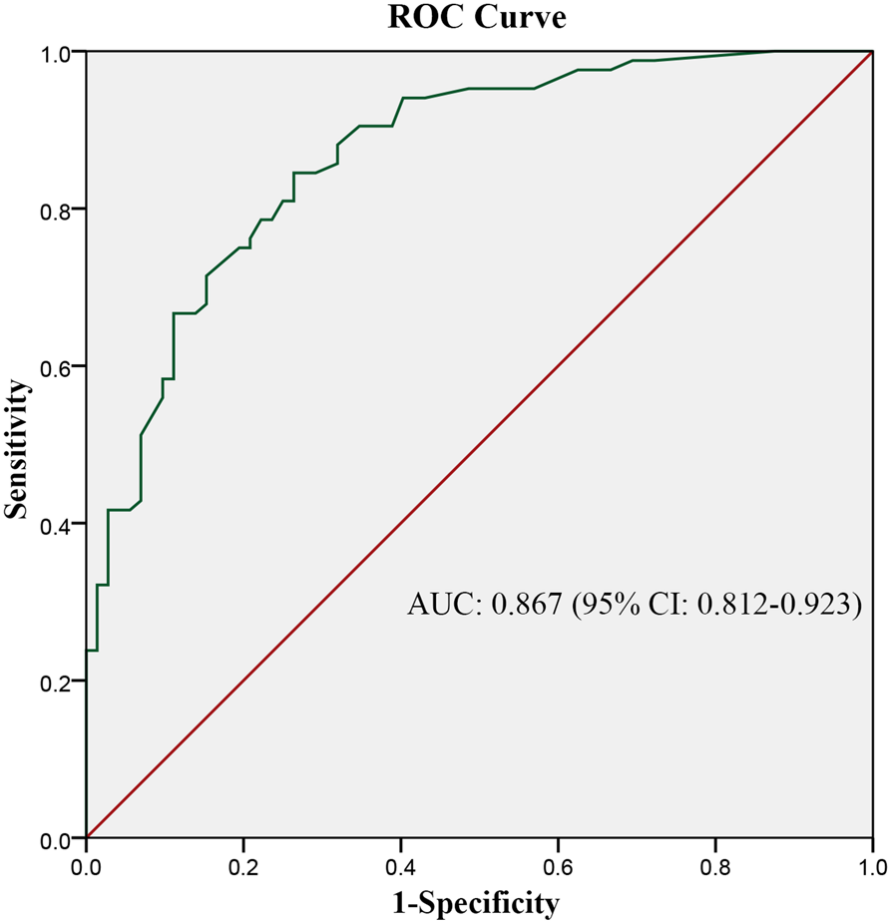
The ROC curve developed for CA recurrence prediction model. The AUC of prediction model was 0.867 (95% CI: 0.812–0.923)

According to the results of multivariate logistic regression analysis, the ROC curve was drawn for the recurrence probability of each CA patient (Fig. 1), and the AUC was 0.867 (95% CI 0.812–0.923). According to the maximum value of Youden index, the corresponding specificity was 73.6% and the sensitivity was 84.5%. Include the prediction equation Logit (*P*) = 0.437.

According to the prediction model equation and Logit (P) value, the following discriminant equation could be obtained:

$${\text{F}}\, = \, - {1}.{2}0{1}\, + \,{1}.{579}*{\text{HPV infection in sexual partners}}\, + \,\,0.{192}*{\text{number of warts}}\, + \,\,\left( { - {1}.{794}*{\text{condom use}}} \right)\, + \,\,{1}.{156}*{\text{urogenital infection}}\,\, + \,\,0.{437}$$

The significance of clinical variable assignment was the same as above. F was the criterion. The calculated value of F could determine whether CA patients have recurrence. When f value ≥ 0, it was judged as recurrence; when f < 0, it was judged as non-recurrence.

### Validation of CA recurrence prediction model

The internal validation was performed with stratified sampling, about 50% of the original cases were included in the above discriminant equation for verification. The results showed that the sensitivity was 80.5% and the specificity was 75.0% (Table 5).

**Table 5:** Internal validation of CA recurrence prediction model

	Recurrence	Non-recurrence	Total
Distinguish recurrence	33	9	42
Distinguish non-recurrence	8	27	35
Total	41	36	77

Sensitivity = 33/(33 + 8)*100% = 80.5%

Specificity = 27/(27 + 9)*100% = 75.0%

From June 2020 to May 2021, 53 CA patients who met the inclusion criteria at the department of dermatological, the First Affiliated Hospital of Harbin Medical University were included in the above discriminant for verification, including 24 cases in the non-recurrence group and 29 cases in the recurrence group, the sensitivity was 75.9% and the specificity was 70.5% (Table 6).

**Table 6:** External validation of CA recurrence prediction model

	Recurrence	Non-recurrence	Total
Distinguish recurrence	22	7	39
Distinguish non-recurrence	7	17	14
Total	29	24	53

Sensitivity = 22/(22 + 7)*100% = 75.9%;

Specificity = 17/(7 + 17)*100% = 70.5%

According to the above results, this model had high stability and predictive value, and the clinical application was simpler. Therefore, this model could be used to predict the probability of CA recurrence in other independent samples.

## Discussion

CA is a common sexually transmitted disease with a high incidence rate. It is a worldwide problem, not only in China, but also in the world. At present, the methods of clinical diagnosis of CA and removal of wart body are clear. The main difficulty is to prevent the recurrence of CA, which often leads to the mental and psychological pressure and heavy economic burden of CA patients [3]. After different treatment methods, the recurrence rate of CA varies. The recurrence rate after 10–25% podophyllum solution treatment is 22–55%, trichloroacetic acid treatment is about 20%, cryotherapy is 22–37%, electroresection is about 60%, surgical resection is about 11%, and interferon treatment is about 40–89% [12]. At present, the common ways to prevent recurrence in clinic include immunotherapy [13], photodynamic therapy [14], topical podophyllin 25–30%, 5-fluorouracil (5-FU) [15], combined traditional Chinese medicine treatment [16] and combined acitretin A [17], etc. Conventional anti-recurrence therapy has greatly reduced the recurrence rate of CA, but these anti-recurrence methods may bring heavy economic burden, and the treatment process is painful, leading to a decline in the treatment confidence of patients. Moreover, the recurrence rate after treatment is 11–89% [12], and the range of recurrence rate is large. In clinical practice, some patients may be over treated, but some patients do not pay attention to anti-recurrence treatment.

### Stay up late is the influencing factor of CA recurrence

This study found that the proportion of stay up late in the relapse group was nearly twice that of the non-relapse group. Stay up late was the influencing factor of CA relapse. Stay up late may weaken the body’s immunity, inhibit the function of T lymphocytes, and weaken the ability to kill and eliminate HPV, leading to the migration of HPV infection and the recurrence of CA [18]. Repeated attacks lead to depression and difficulty in falling asleep, forming a vicious circle [19]. Therefore, to ensure adequate and regular sleep, so that the body into a good state, is conducive to the clean-up of the virus.

### Sexual partners with HPV infection are more likely to relapse

This study found that the rate of HPV infection in sexual partners of patients with CA recurrence group was about three times that of the non-recurrence group, and about 25% of patients in the recurrence group were directly caused by sexual partner infection. Sexual contact transmission is the main transmission route of CA. the infection mechanism is the friction at the sexual contact site, i.e., virus inoculation. HPV virus particles enter the skin fissures of the infected person with the shedding of the infected person’s epithelium to complete the inoculation. After the virus enters the epidermis, it forms a latent infection in the basal layer of keratinocytes. The replication process of infected epidermis allows HPV to enter the nucleus, replicate and spread with cell division, and form warts [20]. As CA infection has an incubation period of 1–6 months [21], when a sexual partner has a skin rash or is accompanied by HPV infection, he should see a doctor in time. In addition, indirect infection is also an important reason for the spread and recurrence of CA, such as sharing bath towels and toilets with CA sexual partners. In clinical practice, many patients have delayed healing and lost confidence in treatment due to repeated attacks, among which there is no lack of knowledge about the mode of transmission of the disease. Therefore, when the sexual partner is accompanied by HPV infection, they should see a doctor in time. The patient and the sexual partner should be treated at the same time. Before the virus is completely removed, both parties should avoid direct and indirect contact.

### Correct use of condom can prevent recurrence of CA

As an effective tool to prevent AIDS, CA and other sexually transmitted diseases, condom can prevent sexually transmitted diseases to a great extent by advocating adherence to and correct use of condoms [22]. In this study, less than one third of the CA patients in the relapse group used condoms correctly, which was much lower than that in the non-relapse group. This will effectively prove that the correct use of condoms can reduce the chances of being infected by the sexual partner even if he or she has HPV infection. The reasons, firstly, the condom is a closed tool, which can effectively prevent the infection of bacteria, viruses and fungi [23]. The second condom is equipped with lubricant, which can reduce local friction during sexual intercourse, so as to reduce the generation of micro wounds and reduce the subclinical infection and colonization of HPV [24]. Thirdly, condoms need to be used correctly. Irregular use of condoms does not reduce the recurrence of CA [25]. Therefore, condoms should not only be used as contraceptives, but also play an indispensable role in reducing the spread of sexually transmitted diseases and preventing the recurrence of diseases. In this study, condom is an independent influencing factor of CA recurrence, which can be used to predict the late recurrence of CA patients. Incorrect use of condom will greatly increase the probability of CA recurrence.

### Patients with genitourinary diseases are prone to CA recurrence

This study found that the proportion of CA patients with urinary tract or genital diseases in the recurrent group (38.1%) was much higher than that in the non recurrent group (11.1%). Female CA patients are often accompanied by gonorrhea, vaginitis, mycoplasma and chlamydia infection. Male patients may also suffer from urethritis and prepuce balanitis. The reason may be that such female patients often have a large number of secretions, resulting in repeated stimulation of the skin and mucosa, reducing the barrier function of the skin and mucosa, resulting in an increase in the probability of HPV colonization in the skin and mucosa [26]. Female CA patients with vaginitis, especially mycotic vaginitis, the probability of recurrence will increase. It may be related to the fact that the body is often in a state of low immune function at the time of the latter's onset, thus promoting the recurrence of CA [27]. Male patients with balanitis prepuce are not uncommon in clinic because of long prepuce. In this study, the proportion of patients with CA combined with prepuce balanitis in the relapse group is higher than that in the non-relapse group. The reason may be that the vulva of patients with prepuce balanitis is often in a warm and humid state, various pathogens accelerate the growth and reproduction, and repeatedly stimulate skin lesions, resulting in prepuce balanitis becoming an influencing factor of CA relapse [28].

### Other diseases caused by HPV infection are the influencing factors of CA recurrence

CA is caused by HPV infection, and HPV infection can also lead to the occurrence of other diseases, including flat warts, filamentous warts, finger/toe warts, Bowenoid papulosis and verrucous epidermal hyperplasia [29]. This study found that the proportion of other diseases associated with HPV infection in the CA recurrence group was significantly higher than that in the non-recurrence group, which proved that the recurrence of CA may be related to the existence of these warts. At present, it has been reported that CA recurrence is related to bowenoid papulosis and seborrheic keratosis [30], but there are few related studies. This study suggests that in clinical diagnosis and treatment, patients with CA should comprehensively check whether there are warts in the trunk and limbs. In order to prevent the recurrence of CA, it is recommended to treat related diseases at the same time.

### The higher the number of warts, the higher the recurrence rate

In addition, through univariate and multivariate analysis, this study concluded that the number of skin lesions was an influencing factor and an independent influencing factor for CA recurrence. The high number of rashes may be related to the imbalance of the patient’s immune system. The decline in immune function leads to a decrease in the inhibition and clearance of HPV by T lymphocytes, resulting in multiple growth of warts [31]. Therefore, when the number of skin lesions in CA patients is large, we should be highly alert to its recurrence, and consciously improve the patient's immunity and enhance the body's ability to clear it.

## Conclusion

In this study, the clinical data of CA patients were analyzed, and age, gender, marriage, education level, smoking, alcoholism, location of wart and HPV type were not related clinical factors for CA recurrence. Stay up late, sexual partner status, condom use, genitourinary disorders, other HPV-infected diseases and the number of warts could affect the recurrence of CA. Among them, sexual partners have HPV infection, the number of warts, condom use and urogenital disorders were the independent influencing factors which caused of CA recurrence. Therefore, when CA patients had the following 4 conditions, the possibility of recurrence should be highly concerned: (1) positive HPV infection in sexual partners; (2) multiple warts; (3) incorrect use of condoms. (4) accompanied by urogenital infection. This study also established a recurrence prediction model for CA patients diagnosed for the first time to provide guidance on whether CA patients need recurrence prevention treatment. At the same time, this study also used cross-validation and external validation to evaluate the stability and application value of the screening model.

## Data Availability

The datasets generated or analyzed during this study are available from the corresponding author on reasonable request.

## Abbreviations

AUA: American Urological Association
CA: Condyloma acuminatum
5-FU: 5-Fluorouracil
HPV: Human Papillomavirus
ROC: Receiver operating characteristic
